# Treatment Cost Along With Pattern of Disease and Therapy in the Intensive Care Unit of a Tertiary Care Teaching Hospital in India

**DOI:** 10.7759/cureus.53052

**Published:** 2024-01-27

**Authors:** Shubham Atal, Ahmad Najmi, Saman Pathan, Saurav Misra, Chenchula Santenna

**Affiliations:** 1 Pharmacology, All India Institute of Medical Sciences (AIIMS) Bhopal, Bhopal, IND; 2 Pharmacology, Kalpana Chawla Government Medical College, Karnal, IND

**Keywords:** ddd, antimicrobials, drug utilization, cost of treatment, icu

## Abstract

Background

The intensive care unit (ICU) represents an important platform for conducting drug utilization analysis using defined daily dose (DDD)/100 bed-days and the financial burden of treatment as patients are seriously ill and are often suffering from chronic critical illnesses. Therefore, in this study, we evaluated the drug utilization patterns and cost of treatment in the ICU.

Methods

A retrospective observational analysis of the medical records obtained for the medical ICU of an apex tertiary care teaching hospital in central India was conducted for a period of three years from 2017 to 2019. All the patients admitted to the medical ICU during the study tenure were included in the study. Patients hospitalized in neonatal intensive care unit (NICU), pediatric intensive care unit (PICU), and surgical ICU were excluded from the study. The socio-demographic and clinical data, utilization of different classes of drugs, WHO-Anatomic Therapeutic Chemical (WHO-ATC) classification, DDD/100 bed days, hospital stay, etc. were analyzed. A partial pharmaco-economic analysis of the average cost of admission to patients was done.

Results

Data from 280 patients was assessed. The mean age was 47 ± 19.18 years and 58% were males. Antibiotics and injections were prescribed to 96% and 97.5% of the patients, respectively, during their ICU stay (median: seven days). Antimicrobial drugs were most frequently prescribed (n=1096, 68%); the most common were beta-lactams and carbapenems, followed by drugs acting on the central nervous system (5%) and cardiovascular system (4.3%). Cefoperazone/sulbactum, ceftriaxone, and piperacillin/tazobactam were the most utilized antibiotics with 8, 16, and 6 DDD/100 bed-days, respectively, while proton pump inhibitors, analgesics, and anti-epileptics were the most frequently prescribed non-antimicrobial drug class. The median cost of treatment per ICU admission was Indian Rupees (INR) 23,347 (IQR 12,552- 65,524).

Conclusion

Drug utilization assessment provides crucial information for understanding the usage of drugs in the settings of the ICU, and should be conducted regularly to help in the proper planning and implementation of rational drug use. Treatment costs reflect the high economic burden seen in ICU admissions.

## Introduction

The intensive care unit (ICU) is a setting where patients are generally given a high number of medications with the majority of them being severely ill and suffering from multiple ailments, resulting in substantial expenditure on hospitalization and drug treatment. In ICU settings, the risk of antimicrobial resistance and adverse events is also significant [1].

Drug utilization research is a basic yet important tool to study the pattern of use of drugs in populations and its impact on the healthcare system. It involves studying various parameters of prescription and use of drugs with special emphasis on the medical and socioeconomic consequences [2,3]. Studies have been conducted across the world including India focusing on different aspects of drug utilization patterns in the ICU, and have shown variable results indicating increased load of drug consumption as well as costs. Most such studies have focused on antibiotic prescription patterns [4,5]. Despite these studies, there is a dearth of data about disease presentations as well as drug prescribing patterns in the ICUs in India, especially in tertiary-level centers in central India. Periodic evaluation of drug utilization in the ICU can be very helpful for the optimization of the healthcare system, proper use of resources, and modifying/monitoring prescription policies [6].

The assumed average maintenance dose per day for a drug used for its primary indication in adults is called the defined daily dose (DDD). The use of DDD is an important tool to compare drug utilization among different clinical setups within a country and between different setups [7,8]. DDD/100 bed-days provides a rough estimate of drug consumption in hospital inpatients and it is a fixed unit of measurement independent of formulation and price. Obtaining data on drug consumption in the ICU in terms of DDD/100 bed-days is very helpful in understanding the local trends, needs, and planning of medical resources in the critical care setting.

An attempt was made to provide an estimate of the economic burden of treatment on the patients admitted to the ICU. A partial pharmaco-economic analysis as a cost of treatment assessment per admission was performed by ascertaining the average costs of drug therapy and investigations borne by the patients [9].

## Materials and methods

This study was conducted retrospectively as a descriptive observational analysis using medical records in the medical ICU of an apex tertiary care teaching hospital in central India (All India Institute of Medical Sciences (AIIMS) Bhopal) for a period of three years from January 2017 to December 2019. Approval was granted by the Institutional Human Ethics Committee, AIIMS Bhopal (approval number: IHEC-LOP/2018/IM0187). All the patients admitted to the medical ICU during the study tenure were included in the study. Patients hospitalized in the neonatal intensive care unit (NICU), pediatric intensive care unit (PICU), and surgical ICU were excluded from the study. Treatment charts of all the patients admitted to the medical ICU during the study period were accessed from the medical record department after seeking due permission.

Data on demographic details, indications of admission (diagnosis), duration of ICU stay, drugs prescribed, laboratory investigations done, patient outcomes, and documented adverse drug reactions (ADRs) were obtained from the records using a predesigned case record form. All data was stored confidentially with access limited only to study investigators. Other standard drug utilization indicators and related parameters (as per WHO) like antimicrobials used per patient, use of fixed-dose combinations (FDCs), generic and branded drugs, oral and parenteral formulations, and outcome of the patients were also calculated. Also, the list of drugs prescribed from the Essential Medical List (EML) and the use of banned drugs were also assessed. For measuring drug consumption, the DDD/100 bed-days was calculated as per the 2010 version of the anatomic therapeutic chemical (ATC)/DDD index, for which bed strength of the ICU and average bed occupancy rate were obtained. DDD was calculated as items issued × amount of drug per item/WHO DDD measure.

A partial pharmaco-economic analysis of the average cost of admission to patients was done for overall ICU admissions. For assessing the cost of treatment, the total cost of drugs per patient per admission was calculated from the price lists obtained from the hospital formulary as well as the average costs obtained from a standard commercial drug directory, Current Index of Medical Specialities (CIMS), India. The total cost of laboratory investigations per admission was calculated from the price list available in the hospital.

Statistical analysis

The data obtained from the medical records was recorded using Microsoft Excel (Microsoft Corporation, Redmond, Washington, United States) on a secure computer. Descriptive statistics was used to display and analyze data through frequencies, percentages, ranges, means/medians, standard deviations, and confidence intervals (95% levels). Statistical analysis was done using IBM SPSS Statistics for Windows, Version 26.0 (Released 2019; IBM Corp., Armonk, New York, United States).

## Results

Demographics and diseased distribution of patients

A total of 280 medical records of patients were analysed thoroughly. Socio-demographic parameters and outcomes among the patients analysed over three years are shown in Table 1. 

**Table 1:** Demographic and outcome parameters among ICU Patients

Demographic and Outcomes	Frequency (N= 280)	Proportion (%)
Gender distribution		
Female	117	41.79
Males	163	58.21
Age distribution		
<18 years	20	7
18-40 years	82	29
41-65 years	103	37
>65 years	75	27
Average length of hospital stay (days)	8.16±6.60 (Median 7, IQR 4-10)	-
Patients with at least one co-morbidity	173	61.79
Outcome of patients		
Death	26	9.29
Patient discharged	249	88.93
Patient discharged against medical advice	5	1.79

The mean age of the patients was 47±19.18 years, with a preponderance of males (58%; n=163). Most patients were in the age group of 41-65 years (37%; n= 103). Out of the 280 patients, 77% were admitted for medical treatment and the rest for surgical treatment. The mean duration of ICU stay was 8.16±6.60 days with the median being seven days (IQR 4-10) with a minimum of one day and a maximum of 51 days.

**Figure 1:**
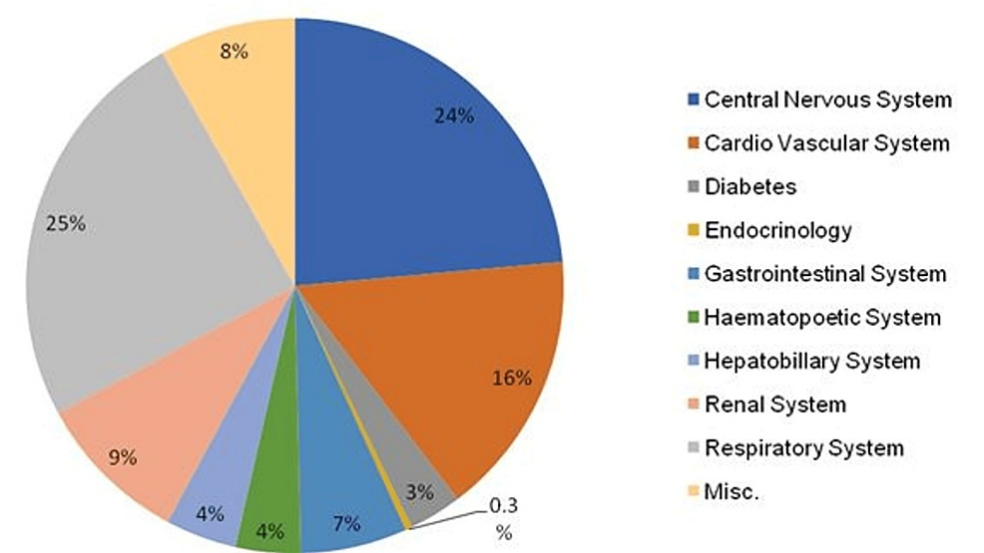
Target organ system involved

The most common diseases/illnesses for which patients were admitted to the ICU were related to the respiratory system (24.6%) and central nervous system (23.5%) followed by the cardiovascular system (16%) (Figure 1).

Of the patients, 65% were admitted with the involvement of more than one system. Some common diseases seen in the patients were pneumonia, exacerbation of chronic obstructive pulmonary disease (COPD)/asthma, coronary artery disease (CAD), cerebrovascular accident (CVA), chronic kidney disease (CKD)/acute kidney injury (AKI), sepsis, etc. Hypertension (17.9%) and diabetes mellitus (16.1%) accounted for the majority of the underlying co-morbid conditions, followed by hypothyroidism, tuberculosis, COPD, liver disease, and cancer.

Prescription pattern

A total of 1612 drugs were prescribed to the patients. Table 2 shows the prescribing pattern and indicators of rational use of drugs using standard indicators.

**Table 2:** Drug prescribing and indicators of rational use EML: Essential Medical List; FDC: Fixed Drug Combination

Prescribing parameters	Frequency (n) and Proportion (%), N = 280
Total drugs prescribed	1612
Average number of medicines per admission (mean ± SD)	5.75 ± 3.48
Encounters with antimicrobials prescribed	1097 (68.05 %)
Medicines prescribed by generic name	890 (55.21 %)
Medicines prescribed from EML	1031 (63.96 %)
Medicines prescribed from FDC	296 (18.36 %)
Encounters with injections prescribed	273 (97.50 %)

The mean number of drugs per prescription was 5.75±3.48. Generic drug prescribing was seen in 55% of drugs (n=890). The percentage of prescribing from the National List of Essential Medicines (NLEM) was 64% whereas antimicrobials and injection prescribing was seen in 96% and 97.5% of the encounters i.e. patient admissions, respectively.

Antimicrobial drugs were most frequently prescribed (n=1096, 68%) including antibacterial (60.5%), antifungal (4.6%), antiviral (2.5%) followed by drugs acting on the CNS (5%), and cardiovascular system (CVS) (4.3%). Out of the total drug formulations analyzed, 74% (n =1197) were given parenterally, 25% (n = 406) were taken orally, and only 1% were either given topically or by inhalation. The prescribing frequency of various drug classes is shown in Table 3.

**Table 3:** Common drug classes prescribed

S. no.	Drug Class	Frequency (n)	Proportion (%)
1	Antibacterial	975	60.55
2	Antifungals	75	4.65
3	Antivirals	40	2.48
4	Antiparasitic	6	0.37
5	Anti-acid secretory	61	3.78
6	Antiemetic	20	1.24
7	Analgesics	43	2.67
8	Cardiovascular drugs	70	4.34
9	Drugs acting on the central nervous system	82	5.08
10	Drugs acting on the respiratory system	49	3.03
11	Anti-thrombotic	64	3.97
12	Hormonal agents	27	1.67
13	Multivitamins	53	3.29
14	Others	47	2.91

DDD/100 bed days

Overall drug utilization in ICU during the study period in terms of DDD/100 bed-days was 10. The most common drugs prescribed are presented in Table 4. Utilization patterns of various drugs, ATC (anatomic, therapeutic, chemical) codes, and DDD/100 bed-days have been shown in Tables 4-5.

**Table 4:** Utilization pattern of various drugs in ICU in DDD/100 bed-days DDD: Defined Daily Dose; ATC Codes: Anatomic, Therapeutic, Chemical Codes; ACE: Angiotensin Converting Enzyme Note: In the ATC classification system, the active substances are classified in a hierarchy with five different levels.

Major Group	Class (ATC Codes)	Total n (%)	Number of DDDs	Number of bed days	DDDs per 100 bed-days
Antimicrobials	Beta lactams (J01D)	316 (28.83)	136.22	1508	9
	Aminoglycosides (J01G)	78 (7.12)	44.47	381	12
	Macrolides (J01F)	107 (9.76)	107.03	467	23
	Quinolones (J01M)	18 (1.64)	17.77	79	22
	Antiparasitic (P01B)	3 (0.27)	16.33	11	148
	Antihelmintics (P02C)	3 (0.27)	3.30	4	83
	Amphenicols (J01B)	8 (0.73)	2.02	50	4
	Antifungals (J02A)	75 (6.84)	124.68	406	31
	Antitubercular (J04A)	13 (1.19)	10.18	69	15
	Carbepenems (J01DH)	144 (13.14)	61.64	999	6
	Imidazoles (J01XD)	42 (3.83)	12.34	218	6
	Penicillins (J01CE)	1 (0.09)	0.00	12	0
	Polymixin (J01X)	40 (3.65)	3.11	262	1
	Sulfamethoxazole + Trimethoprim (J01EE)	6 (0.55)	2.90	29	10
	Tetracyclines (J01AA)	64 (5.84)	59.09	361	16
	Glycopeptides (J01XA)	99 (9.76)	83.62	445	19
	Antivirals (J05A)	40 (3.65)	1037.36	190	546
	Other Antimicrobials (J01X)	40 (3.65)	33.26	198	17
	Total	1097 (100)	1755.31	5689.00	967.53
Drugs acting on anti-acid secretary system	Proton pump inhibitors (A02B)	57 (96.61)	53	351	15
	H2 blockers (A02B)	2 (3.39)	0.17	23	1
	Total	59 (11.46)	53.17	374	16
Analgesic	Non-steroidal anti inflammatory drugs (NSAIDs) (N02B)	31 (71.09)	10	139	7
	Opioid Analgesic (N02A)	11 (25.58)	2.1	30	7
	Opioids and Non opioid analgesics (N02AJ)	1 (2.32)	0.09	1	9
	Total	43 (8.35)	12.19	170	23
Cardiovascular drugs	Beta blockers (C07A)	13 (22.41)	9.18	70	13
	Diuretics (C03C)	23 (39.65)	7.23	121	6
	Calcium channel blockers (C08C)	18 (31.03)	101.83	96	106
	ACE inhibitors (C09A)	4 (6.89)	4.00	23	17
	Total	58 (11.26)	122.24	310	123
Drugs acting on central nervous system	Anti-epileptics (N03A)	30 (76.92)	23.25	202	12
	Antipsychotics (N05B)	7 (17.95)	3.09	49	6
	Psycho-analeptics (N06B)	2 (5.13)	1.54	17	9
	Total	39 (7.57)	27.88	268	27

**Table 5:** Utilization pattern of various antimicrobials in the ICU DDD: Defined Daily Dose; ATC Codes: Anatomic, Therapeutic, and Chemical Codes Note: In the ATC classification system, the active substances are classified in a hierarchy with five different levels.

Drugs	ATC Codes	Number of DDDs	DDD/100 bed days
Piperacillin + Tazobactam	J01CR05	14	6.04
Meropenem	J01DH02	3	6.18
Teicoplanin	J01XA02	0.4	22.52
Cefoparazone + Sulbactam	J01DD62	4	8.45
Amikacin	J01GB06	1	12.66
Ceftriaxone	J01DD04	2	16.00
Azithromycin	J01FA10	0.3	46.66
Clindamycin	J01FF10	1.8	6.90
Metronidazole	J01XD01	1.5	5.67
Imipenem	J01DH51	2	5.92
Doxycycline	J01AA02	0.1	22.53
Oseltamivir	J05AH02	0.15	27.42
Tigecycline	J01AA12	0.1	13.58
Amoxycillin + Clavulanic acid	J01CR02	3	6.94
Amphotericin B	J02AA01	0.04	35.06
Vancomycin	J01XA01	2	8.91
Linezolid	J01XX08	1.2	12.87
Caspofungin	J02AX04	0.05	21.00
Erythromycin	J01FA01	1	5.88

Cefoperazone/sulbactam, ceftriaxone, and piperacillin/tazobactam were the most utilized antimicrobials with eight, 16, and six DDD/100 bed-days, respectively. Antimicrobials with the highest DDD/100 bed days were azithromycin (46), amphotericin B (35), oseltamivir (27), and teicoplanin (22.5). Proton pump inhibitors, analgesics, and antiepileptics were the most highly utilized non-antimicrobial drug classes with 15, 23, and 12 DDD/100 bed-days, respectively. The most common non-antimicrobial drug consumption in the number of DDD/100 bed days and ATC codes are presented in Table 6.

**Table 6:** Common non-antimicrobial consumption presented as DDD/100 bed-days DDD: Defined Daily Dose; ATC Codes: Anatomic, Therapeutic, and Chemical Codes

Major groups	Drugs	ATC	DDD in gm	DDD/100 bed days
Proton pump Inhibitor	Pantoprazole	A02BC02	0.04	15.00
Analgesic	Diclofenac	M01AB05	0.1	32.14
	Diclofenac+ serratiopeptidase	M01AB05	0.1	12.00
	Morphine	N02AA01	0.03	5.00
	Fentanyl	N02AB03	1.2	0.03
	Paracetamol + Tramadol	N01AJ13	2	4.50
	Tramadol	N02AX02	0.3	11.11
	Paracetamol	N02BE01	3	5.28
Antiemetic	Ondansetron	A044401	0.016	313.73
Diuretic	Furosemide	C03CA01	0.04	14.32
	Torasemide	C03CA04	0.015	1.74
	Furosemide + Spironolactone	C03EB01	1	1.00
	Mannitol	R05CB16	0.8	1.32
	Metolazone	V03AE07	6	0.01
Antiplatlets	Aspirin	B01AC06	1	3.45
	Clopidogrel	B01AC04	0.75	7.41
Steroids	Dexamethasone	H02AB02	0.0015	62.63
	Hydrocortisone	H02AB02	0.3	81.43
	Methylprednisolone	H02AB04	0.02	460.0
	Prednisolone	H02AB06	0.01	33.16
Antiepileptic	Clonazepam	N03AE01	0.008	46.88
	Levetiracetam	N03AX14	1.5	5.44
	Phenytoin	N03AB02	0.3	9.73
	Valproic acid	N03AG01	1.5	4.39

Non-antimicrobials with the highest DDD/100 bed days were methylprednisolone (460.0), ondansetron (313.7), hydrocortisone (81.43), and dexamethasone (62.63).

Antibiotic sensitivity testing

**Figure 2:**
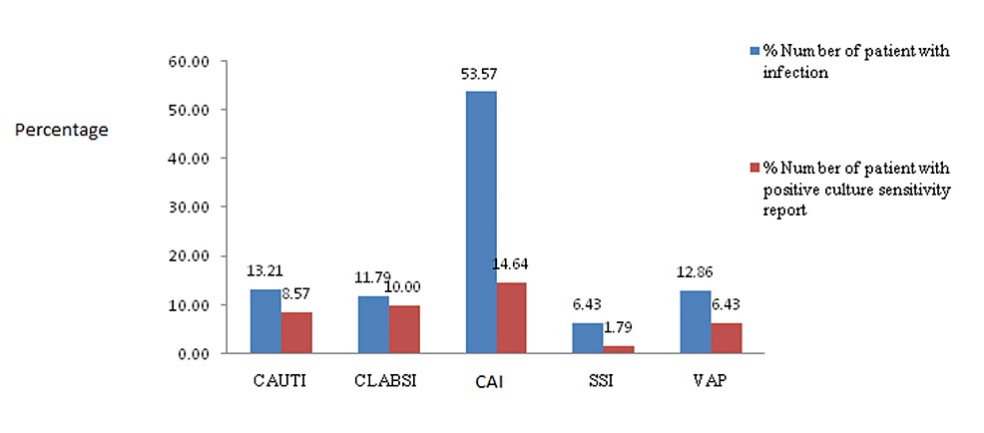
Type of Infections in the ICU CAUTI: Catheter-Associated Urinary Tract Infections; CLABSI: Central Line-Associated Bloodstream Infection; CAI: Community-Acquired Infection; SSI: Surgical Site Infection; VAP: Ventilator-Associated Pneumonia

The most common type of infections seen were community-acquired infection (CAI) (53.6%; n=150) followed by catheter-associated urinary tract infection (CAUTI) (13.2%; n=37) and ventilator-associated pneumonia (VAP) (n=36; 12.9%) as shown in Figure 2.

Antibiotic sensitivity testing was performed in 97.8% of the patients. A total of 40 organisms were isolated. The major isolated organisms were *Klebsiella pneumoniae, * *Acinetobacter baumannii, Staphylococcus aureus, * budding yeast cell, and *Citrobacter *spp (0.4%) as shown in Tables 7-8. 

**Table 7:** Type of Infections in the ICU Some patients have more than one infection MRCoNS: Methicillin-Resistant Coagulase Negative *Staphylococci*; CAUTI: Catheter-Associated Urinary Tract Infection; CLABSI: Central-Line Associated Bloodstream Infection; CAI: Community-Acquired Infection; SSI: Surgical Site Infection; VAP: Ventilator-Associated Pneumonia

Microorganisms	CAUTI N (%)	CLABSI N(%)	CAI N(%)	SSI N(%)	VAP N (%)	Total N(%)
3 Morphotypes	2 (0.81)	0 (0.00)	2 (0.81)	0 (0.00)	1 (0.40)	5 (2.02)
Acinetobacter baumannii	5 (2.02)	14 (5.65)	14 (5.65)	3 (1.21)	3 (1.21)	39 (15.73)
Acinetobacter haemolysis	0 (0.00)	1 (0.40)	0 (0.00)	0 (0.00)	0 (0.00)	1 (0.40)
*Acinetobacter *Spp.	0 (0.00)	0 (0.00)	2 (0.81)	0 (0.00)	2 (0.81)	4 (1.61)
Budding yeast cell	4 (1.61)	3 (1.21)	5 (2.02)	0 (0.00)	2 (0.81)	14 (5.65)
Candida albicans	5 (2.02)	2 (0.81)	0 (0.00)	0 (0.00)	0 (0.00)	7 (2.82)
Candida parapsilosis	0 (0.00)	1 (0.40)	0 (0.00)	0 (0.00)	0 (0.00)	1 (0.40)
*Candida *Spp.	2 (0.81)	1 (0.40)	1 (0.40)	0 (0.00)	2 (0.81)	6 (2.42)
Chikunguniya	0 (0.00)	0 (0.00)	1 (0.40)	0 (0.00)	0 (0.00)	1 (0.40)
Chysobacterium indologenes	0 (0.00)	0 (0.00)	1 (0.40)	0 (0.00)	0 (0.00)	1 (0.40)
*Citrobacter *Spp.	0 (0.00)	1 (0.40)	0 (0.00)	0 (0.00)	0 (0.00)	1 (0.40)
Escherichia coli	6 (2.42)	6 (2.42)	9 (3.63)	1 (0.40)	1 (0.40)	23 (9.27)
Enterococcus faecalis	0 (0.00)	0 (0.00)	0 (0.00)	0 (0.00)	1 (0.40)	1 (0.40)
Enterococcus faecium	0 (0.00)	0 (0.00)	3 (1.21)	0 (0.00)	0 (0.00)	3 (1.21)
*Enterococcus *Spp.	3 (1.21)	5 (2.02)	3 (1.21)	0 (0.00)	0 (0.00)	11 (4.44)
Enterovirus	0 (0.00)	1 (0.40)	0 (0.00)	0 (0.00)	0 (0.00)	1 (0.40)
Gram-negative bacilli	1 (0.40)	0 (0.00)	2 (0.81)	0 (0.00)	3 (1.21)	6 (2.42)
Gram-negative cocci bacilli	0 (0.00)	1 (0.40)	0 (0.00)	0 (0.00)	0 (0.00)	1 (0.40)
Gram-positve cocci bacilli	0 (0.00)	0 (0.00)	0 (0.00)	0 (0.00)	2 (0.81)	2 (0.81)
Gram-positive bacilli	0 (0.00)	0 (0.00)	2 (0.81)	0 (0.00)	1 (0.40)	3 (1.21)
Gram-positive cocci	3 (1.21)	0 (0.00)	0 (0.00)	0 (0.00)	1 (0.40)	4 (1.61)
H1N1	0 (0.00)	2 (0.81)	1 (0.41)	0 (0.00)	3 (1.21)	6 (2.42)
HBV	0 (0.00)	0 (0.00)	1 (0.40)	0 (0.00)	0 (0.00)	1 (0.40)
Influenza A	1 (0.40)	0 (0.00)	0 (0.00)	0 (0.00)	0 (0.00)	1 (0.40)
Insignificant bacterial growth	2 (0.81)	0 (0.00)	1 (0.40)	0 (0.00)	0 (0.00)	3 (1.21)
Insignificant bacteriuria	2 (0.81)	1 (0.40)	4 (1.61)	0 (0.00)	0 (0.00)	7 (2.82)
Klebsiella pneumoniae	6 (2.42)	15 (6.05)	12 (4.84)	2 (0.81)	6 (2.42)	41 (16.53)
*Klebsiella *Spp.	0 (0.00)	2 (0.81)	0 (0.00)	0 (0.00)	0 (0.00)	2 (0.81)
Methicillin-sensitive* Staphylococcus aureus *(MSSA)	0 (0.00)	0 (0.00)	1 (0.40)	0 (0.00)	0 (0.00)	1 (0.40)
MRCoNS* Staphylococcus aureus*	4 (1.61)	12 (4.84)	2 (0.81)	2 (0.81)	2 (0.81)	22 (8.87)
MRCoNS* Staphylococcus epidermitis*	0 (0.00)	1 (0.40)	0 (0.00)	0 (0.00)	0 (0.00)	1 (0.40)
MRCoNS* Staphylococcus spp*	0 (0.00)	0 (0.00)	2 (0.81)	0 (0.00)	0 (0.00)	2 (0.81)
Methicillin-resistent *Staphylococcus aureus *(MRSA)	0 (0.00)	1 (0.41)	0 (0.00)	0 (0.00)	0 (0.00)	1 (0.40)
Non Albicans candida	2 (0.81)	2 (0.81)	1 (0.40)	0 (0.00)	0 (0.00)	5 (2.02)
Proteus mirabilis	0 (0.00)	0 (0.00)	1 (0.40)	0 (0.00)	0 (0.00)	1 (0.40)
Pseudomonas aeruginosa	0 (0.00)	2 (0.81)	1 (0.40)	2 (0.81)	3 (1.21)	8 (3.23)
*Pseudomonas *Spp.	2 (0.81)	2 (0.81)	2 (0.81)	0 (0.00)	1 (0.40)	7 (2.82)
Skin Contaminants	1 (0.40)	0 (0.00)	0 (0.00)	0 (0.00)	0 (0.00)	1 (0.40)
Staphylococcus aureus	0 (0.00)	0 (0.00)	0 (0.00)	1 (0.40)	0 (0.00)	1 (0.40)
Stenotrophomonas maltophillia	0 (0.00)	1 (0.40)	0 (0.00)	0 (0.00)	0 (0.00)	1 (0.40)
Streptococcus viridans	0 (0.00)	0 (0.00)	1 (0.40)	0 (0.00)	0 (0.00)	1 (0.40)
Total	51 (20.56)	77 (31.05)	75 (30.24)	11 (4.44)	34 (13.71)	248 (100)

**Table 8:** Causative microorganism according to site of infection Patients may have more than one infection site or microorganism MRSA: Methicillin-Resistant *Staphylococcus aureus*; MRCoNS: Methicillin-Resistant Coagulase Negative *Staphylococcus*

Microorganisms	Blood, n (%)	Respiratory, n (%)	Gastrointestinal, n (%)	Urine, n (%)	Throat, n (%)	Wound/Pus, n (%)	Total, n (%)
3 Morphotypes	1 (0.40)	1 (0.40)	0 (0.00)	2 (0.81)	0 (0.00)	1 (0.40)	5 (2.02)
Acinetobacter baumannii	6 (2.42)	25 (10.08)	1 (0.40)	2 (0.81)	2 (0.81)	3 (1.21)	39 (15.73)
Acinetobacter haemolysis	1 (0.40)	0 (0.00)	0 (0.00)	0 (0.00)	0 (0.00)	0 (0.00)	1 (0.40)
*Acinetobacter *Spp.	0 (0.00)	4 (1.64)	0 (0.00)	0 (0.00)	0 (0.00)	0 (0.00)	4 (1.61)
Budding yeast cell	3 (1.21)	3 (1.21)	0 (0.00)	7 (2.82)	1 (0.40)	0 (0.00)	14 (5.65)
Candida albicans	2 (0.81)	2 (0.81)	0 (0.00)	3 (1.21)	0 (0.00)	0 (0.00)	7 (2.82)
Candida parapsilosis	1 (0.40)	0 (0.00)	0 (0.00)	0 (0.00)	0 (0.00)	0 (0.00)	1 (0.40)
*Candida *Spp*.*	0 (0.00)	1 (0.40)	0 (0.00)	5 (2.02)	0 (0.00)	0 (0.00)	6 (2.42)
Chikunguniya	1 (0.40)	0 (0.00)	0 (0.00)	0 (0.00)	0 (0.00)	0 (0.00)	1 (0.40)
Chysobacterium indologenes	1 (0.40)	0 (0.00)	0 (0.00)	0 (0.00)	0 (0.00)	0 (0.00)	1 (0.40)
*Citrobacter *Spp*.*	1 (0.40)	0 (0.00)	0 (0.00)	0 (0.00)	0 (0.00)	0 (0.00)	1 (0.40)
Escherichia coli	7 (2.82)	5 (2.05)	0 (0.00)	8 (3.23)	0 (0.00)	3 (1.21)	23 (9.27)
Enterococcus faecalis	0 (0.00)	0 (0.00)	0 (0.00)	1 (0.40)	0 (0.00)	0 (0.00)	1 (0.40)
Enterococcus faecium	1 (0.40)	0 (0.00)	0 (0.00)	2 (0.81)	0 (0.00)	0 (0.00)	3 (1.21)
*Enterococcus *Spp*.*	4 (1.61)	5 (2.02)	1 (0.40)	1 (0.40)	0 (0.00)	0 (0.00)	11 (4.44)
Enterovirus	1 (0.40)	0 (0.00)	0 (0.00)	0 (0.00)	0 (0.00)	0 (0.00)	1 (0.40)
Gram-negative bacilli	1 (0.40)	2 (0.81)	1 (0.40)	1 (0.40)	1 (0.40)	0 (0.00)	6 (2.42)
Gram-negative cocci bacilli	0 (0.00)	1 (0.40)	0 (0.00)	0 (0.00)	0 (0.00)	0 (0.00)	1 (0.40)
Gram-positve cocci bacilli	0 (0.00)	0 (0.00)	0 (0.00)	0 (0.00)	2 (0.81)	0 (0.00)	2 (0.81)
Gram-positive bacilli	2 (0.81)	0 (0.00)	0 (0.00)	0 (0.00)	1 (0.40)	0 (0.00)	3 (1.21)
Gram-positive cocci	1 (0.40)	0 (0.00)	0 (0.00)	1 (0.40)	2 (0.81)	0 (0.00)	4 (1.61)
H1N1	0 (0.00)	1 (0.40)	0 (0.00)	0 (0.00)	5 (2.02)	0 (0.00)	6 (2.42)
HBV	0 (0.00)	0 (0.00)	0 (0.00)	0 (0.00)	1 (0.40)	0 (0.00)	1 (0.40)
Influenza A	0 (0.00)	0 (0.00)	0 (0.00)	0 (0.00)	1 (0.40)	0 (0.00)	1 (0.40)
Insignificant bacterial growth	0 (0.00)	1 (0.42)	0 (0.00)	2 (0.81)	0 (0.00)	0 (0.00)	3 (1.21)
Insignificant bacteriuria	1 (0.40)	1 (0.40)	0 (0.00)	5 (2.02)	0 (0.00)	0 (0.00)	7 (2.82)
Klebsiella pneumoniae	14 (5.65)	16 (6.45)	0 (0.00)	3 (1.21)	4 (1.61)	4 (1.61)	41 (16.53)
*Klebsiella *Spp*.*	1 (0.40)	1 (0.40)	0 (0.00)	0 (0.00)	0 (0.00)	0 (0.00)	2 (0.81)
Methicillin-sensitive* Staphylococcus aureus*	0 (0.00)	0 (0.00)	0 (0.00)	0 (0.00)	0 (0.00)	1 (0.40)	1 (0.40)
MRCoNS* Staphylococcus aureus*	21 (8.47)	1 (0.40)	0 (0.00)	0 (0.00)	0 (0.00)	0 (0.00)	22 (8.87)
MRCoNS* Staphylococcus epidermitis*	1 (0.40)	0 (0.00)	0 (0.00)	0 (0.00)	0 (0.00)	0 (0.00)	1 (0.40)
MRCoNS* Staphylococcus spp*	2 (0.81)	0 (0.00)	0 (0.00)	0 (0.00)	0 (0.00)	0 (0.00)	2 (0.81)
MRSA	1 (0.40)	0 (0.00)	0 (0.00)	0 (0.00)	0 (0.00)	0 (0.00)	1 (0.40)
Non Albicans candida	1 (0.40)	1 (0.40)	0 (0.00)	3 (1.21)	0 (0.00)	0 (0.00)	5 (2.02)
Proteus mirabilis	0 (0.00)	0 (0.00)	0 (0.00)	1 (0.40)	0 (0.00)	0 (0.00)	1 (0.40)
Pseudomonas aeruginosa	3 (1.21)	4 (1.61)	1 (0.40)	0 (0.00)	0 (0.00)	0 (0.00)	8 (3.23)
*Pseudomonas *Spp.	4 (1.61)	2 (0.81)	0 (0.00)	1 (0.40)	0 (0.00)	0 (0.00)	7 (2.82)
Skin Contaminants	1 (0.40)	0 (0.00)	0 (0.00)	0 (0.00)	0 (0.00)	0 (0.00)	1 (0.40)
Staphylococcus aureus	1 (0.40)	0 (0.00)	0 (0.00)	0 (0.00)	0 (0.00)	0 (0.00)	1 (0.40)
Stenotrophomonas maltophillia	0 (0.00)	1 (0.40)	0 (0.00)	0 (0.00)	0 (0.00)	0 (0.00)	1 (0.40)
Streptococcus viridans	1 (0.40)	0 (0.00)	0 (0.00)	0 (0.00)	0 (0.00)	0 (0.00)	1 (0.40)
Total	86 (34.68)	78 (31.45)	4 (1.61)	48 (19.35)	20 (8.06)	12 (4.84)	248 (100)

**Figure 3:**
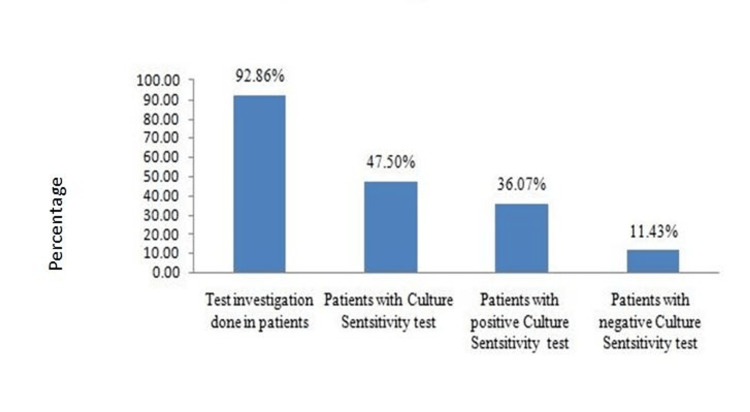
Culture and Sensitivity Test

Patients with infections and positive culture sensitivity reports are shown in Figure 3.

Cost analysis

The median total cost of drugs was Indian Rupee (INR) 15,919.26 (interquartile Range (IQR) 6086-53,031) per patient while ranging from INR 26 to INR 581,963. This cost was generated by taking the generic cost of all the medicines prescribed, taken from the ‘Jan-Aushadhi’ store (under the Government of India's generic drug scheme) in the hospital. The median total cost of laboratory investigations per patient was INR 720 (IQR 374-1284) per patient. The main investigations that contributed to this cost were cell blood count, renal function test, liver function test, electrolytes, culture sensitivity test, and CRP in 84%, 85%, 77%, 85%, 46%, and 60% of patients, respectively. The per day ICU stay cost was fixed at Rs. 1000. The median daily cost of ICU stay per patient was INR 7000 (IQR 4000-9000). The total direct median cost of treatment comprising medicines, investigations, and hospital stay charges over an average ICU admission was INR 23,347 (IQR 12,552- 65,524) of which medicines contributed to 85%, and the rest was contributed by ICU stay charges and laboratory investigation costs.

## Discussion

A thorough analysis of 280 ICU records was conducted over a three-year period at our apex tertiary care center to evaluate the overall picture of admitted patients, drug prescribing/utilization patterns, and cost analysis. The results were consistent with the patterns anticipated at the tertiary care level. A higher proportion of male patients was admitted to the ICU (58%), which is comparable to other Indian studies conducted at tertiary care centers in different regions of the country [10]. The majority of patients were in the age group of 41-65 years with a mean age of 47±19.18 years; this age distribution was different from studies conducted in other parts of India [11]. The average age of patients was higher in our study than in other studies; a longer stay may be related to more elderly admissions. The median duration of stay of seven days in the ICU is on the higher side compared to other studies [12,13]. The financial burden on the patient increases with the length of stay in the ICU. The median cost of treatment per person in our study of INR 23347 is also correspondingly higher than that found in comparable studies conducted in India and Nepal [14,15]. This can be attributed to the fact that more patients in serious conditions spent longer time in our ICU and required more medications and investigations. The majority of the expense is attributable to the antimicrobials prescribed, and it is known that the practice of overprescribing frequent, broad-spectrum antibiotics is rather common [2,6]. However, a rationality assessment was not done formally in this analysis. However, the cost of treatment was lower than the study conducted by Patel et al. from India [6] and Inan et al. from Turkey [16]. The variation in the type and severity of indications of conditions in admitted patients, the different prescribing patterns, or currency differences may be the cause of the variations in the average cost of treatment between these analyses. In our study, 89% of patients were discharged and discharge was against medical advice in only 2% of cases, which is less than that reported in another analysis from Kerela, India [17].

The average number of drugs per prescription (ICU stay) in our study was 8.16±6.60. This ‘polypharmacy’ is justified in the ICU setup of a tertiary care facility equipped to handle patients with severe illnesses, numerous co-morbidities, or challenging or non-responsive conditions. However, this was relatively less compared to many studies such as the ones conducted by Pandiamunian et al. (10.4±2 drugs) [17] and Smythe et al. (12±7.6 drugs) [18], while being slighter higher than that by Inan et al. (eight drugs) [16] and Shobha et al. (6.9 drugs) [13]. This is a positive indicator suggesting that overprescribing is perhaps not prevalent in our setting. Nevertheless, it is still advisable to be vigilant about the rational use of drugs to limit any unnecessary drug prescribing to lower the risk of drug interactions, hospital costs, and the emergence of bacterial resistance.

The proportion of prescriptions with antimicrobials (68%) and injections (98%) was relatively higher than some other published studies [19,20], though this needs to be explored. Because ICU patients are 5-10 times more likely to develop nosocomial infections, a high rate of antibiotic prescriptions is possibly justified with prophylactic administration of drugs to stop the development and spread of infections. Generic prescribing and prescribing from ELM is recommended by the WHO to be 100%, but it was significantly lesser in the prescriptions analyzed, 55% and 64%, respectively. There is a definite scope for more in-depth analysis of this as the national and hospital EMLs should be ideally adhered to even in tertiary-level prescribing. However, the complexity of cases may necessitate the use of medications that are not listed yet in ELM lists, which also suggests a possible review of the lists particularly at the institutional level.

Antimicrobial utilization pattern

Antimicrobials were the most frequently prescribed drugs in the analysis with an average of 3.5 antimicrobials administered per patient. These results are comparable to previous literature which indicates a range of 1.73-5.1 as reported from various geographic regions and patient types [21,22]. The most common antibacterial drug classes prescribed in our ICU were beta-lactams (29%), carbapenems (13%), macrolides (10%), and glycopeptides (10%). In other studies, cephalosporins (73.30%), metronidazole (55.67%), and penicillins were the most commonly prescribed antimicrobials [23,24]. Among individual drugs, the piperacillin+tazobactam combination (7%) was the most common, followed by meropenem (6%) and teicoplanin (5%). This was quite variable at other centers [23,24]. The antibiotic use policies and changes in culture sensitivity patterns may be responsible for such differences in utilization patterns. All these factors including policies, prescription audits, and culture sensitivity patterns should ideally be regularly and minutely assessed. This study gains strength by including a diverse patient population from the ICU of an apex tertiary care hospital.

A high proportion of patients (96%) received at least one antimicrobials prescription, which is in line with the findings by Shankar et al. [2], but higher than other studies [11,22,24,25], thus revealing that, depending on the health center, the percentage of patients receiving at least one antimicrobial varied from 41% to 98%. Since the patients across these studies are not matched, no firm conclusions can be made.

Quantitatively, in terms of DDD/100 bed-days, azithromycin (46.66), doxycycline (22.53), teicoplanin (22.52), and ceftriaxone (16) were the most frequently used antimicrobials in our study. Comparatively, the DDD/100 bed-days for the six most commonly prescribed antibiotics were much lesser for doxycycline (8.7), piperacillin/tazobactam (7.8), ceftriaxone (6.2), azithromycin (6.1), metronidazole (5.1), and meropenem (4.8) in a study by Marasine et al. [26]. In another study conducted in western Nepal, penicillin, fluoroquinolones, second-generation cephalosporins, and third-generation cephalosporins were used at rates of 55.1, 5.34, 0.82, and 13.74 DDD/100 bed-days respectively [27]. A similar study from India found that third-generation cephalosporins (18.48), meropenem (16.47), levofloxacin (15.97), metronidazole (14.65), and ceftriaxone (13.42) were the five antimicrobials that were most frequently used [24].

Antibiotic sensitivity testing

We found that *Klebsiella pneumonia,* *Acinetobacter baumannii*, *Citrobacter *spp*.,* and *Staphylococcus aureus *were the most frequently isolated microorganisms at our center. *Klebsiella *was also one of the most frequently isolated organisms in the study by Patel et al. [6], whereas *Pseudomonas,* *Escherichia coli*, and *S. aureus *have been mentioned in other studies [28]. Almost all (97.5%) patients underwent antibiotic sensitivity in the current study, but other studies indicated that sensitivity testing was used less frequently [28]. If there is underutilization of antibiotic sensitivity testing, it can be challenging to ensure the rational use of antibiotics for admitted patients. To improve the selection of antimicrobials and lower mortality, the use of antibiotic sensitivity testing is imperative. At the same time, avoiding the use of unnecessary antibiotics helps to improve patient-related outcomes and reduces costs too.

ICU treatment costs

The median cost per prescription was INR 23,347 (IQR 12,552- 65,524). This is very high, especially in our setting where most of the patients are from the lower socioeconomic strata and cannot afford relatively high out-of-pocket expenditure on treatment. However, government schemes like Ayushman Bharat in India have come to patients’ rescue in this regard. This is a national public health insurance scheme of the Government of India that aims to provide free access to health insurance coverage for low-income earners (roughly 50% of the population) in the country, thus saving them from having to spend high costs on their medical treatment [29,30]. Analysis of the data revealed that a significant portion of drug cost was driven by antimicrobials. This is probably because, in an ICU setting, there is a justified need to give extended-spectrum antibiotics as empirical therapy, which is usually expensive. An area of concern was the lower proportion of drugs prescribed as generics (only 55%). There are several benefits of prescribing generic drugs including increased patient compliance and lower cost of drug therapy [29,30]. In India, ICU costs and outcomes have received attention previously. In previous studies, ICU costs ranged from INR 1,973 per patient per day in a public hospital to INR 10,364 per day [29,30]. A bulk of the patients (75%) in the current study belonged to the middle socioeconomic status, with about 40% admitted with poisoning and febrile illnesses occurring in relatively younger patients (mean age, 42 years) with equal rural and urban distribution. These factors may also affect ICU costs and affordability.

Limitations

The study had some limitations. This was a single-center study, reflective of practices at this center but it can be extrapolated to other similar centers in the country. In cost analysis, only direct medical cost was included, not indirect medical cost, this being a retrospective analysis. Costing was done as per the cost applicable at the time of the study, which may be different from the current cost.

## Conclusions

ICU patients require a high average number of medications during their admission, with antimicrobial medications being most commonly prescribed followed by anti-acid secretory drugs. Multiple system involvement was commonly seen along with the presence of co-morbidities. All of these factors lead to a relatively high median cost of treatment which reached approximately INR 23,000 over an average stay of seven days in the ICU. Regular audits and reviews of antimicrobial policy are recommended to curtail the polypharmacy and inappropriate use of antibiotics. Since the drug cost was mostly due to the prescription of extended-spectrum antibiotics, the hospital pharmacy should be encouraged to procure more cost-effective alternative antibiotics in the future.
